# Supplementary material for: Validation of the Reference Genes for Expression Analysis in the Hippocampus after Transient Ischemia/Reperfusion Injury in Gerbil Brain
Source: Int J Mol Sci. 2023 Feb 1;24(3):2756. doi: 10.3390/ijms24032756 (PMC9917415; doi:10.3390/ijms24032756)
Supplement: Supplementary file 1 [file ijms-24-02756-s001.zip › ijms-2087448-supplementary.pdf]

Table S1. Mean Ct values and standard deviation (SD) of candidate reference genes in the CA1 and CA2-3, DG areas of the hippocampus at several time points after 5 min of ischemia. Data are expressed as mean ± SD, n = 30.

Gene	mean Ct	SD
Gapdh	21.42	0.77
Actb	21.49	0.84
18SrRNA	12.28	1.16
Hprt1	26.36	0.7
Hmbs	30.23	0.59
Ywhaz	23.21	0.85
Bud23	29.03	0.69

Table S2. Stability value of candidate reference genes from RefFinder and each algorithm from the CA1 and CA2-3,DG regions of the hippocampus, separately. SV and R stand for stability value and rank for each method, respectively.

Region of hippocampus	Gene name	RefFinder		Delta Ct		BestKeeper		NormFinder		geNorm	
		SV	R	SV	R	SV	R	SV	R	SV	R
CA1	*Gapdh*	1.861	2	0.519	1	0.562	4	0.177	1	0.31	3
	Actb	2.06	3	0.548	3	0.436	2	0.325	3	0.276	1
	18SrRNA	7.00	7	1.048	7	1.102	7	0.981	7	0.658	7
	Hprt1	5.733	6	0.747	6	0.576	5	0.595	6	0.501	6
	Hmbs	1.414	1	0.538	2	0.383	1	0.271	2	0.276	1
	Ywhaz	4.949	5	0.612	5	0.673	6	0.35	4	0.417	5
	Bud23	3.936	4	0.591	4	0.56	3	0.389	5	0.326	4
CA2-3,DG	*Gapdh*	3.936	5	0.58	4	0.556	5	0.346	4	0.417	3
	Actb	6.735	7	0.834	7	0.671	6	0.74	7	0.643	7
	18SrRNA	6.236	6	0.806	6	0.824	7	0.699	6	0.567	6
	Hprt1	1.189	1	0.543	1	0.416	1	0.278	2	0.348	1

	Hmbs	2.213	2	0.546	2	0.521	3	0.241	1	0.446	4
	Ywhaz	2.659	3	0.62	5	0.503	2	0.444	5	0.348	1
	Bud23	3.663	4	0.574	3	0.536	4	0.31	3	0.473	5
